# Predictive model of sperm whale prey capture attempts from time-depth data

**DOI:** 10.1186/s40462-023-00393-2

**Published:** 2023-06-08

**Authors:** Sergi Pérez-Jorge, Cláudia Oliveira, Esteban Iglesias Rivas, Rui Prieto, Irma Cascão, Paul J. Wensveen, Patrick J. O. Miller, Mónica A. Silva

**Affiliations:** 1 Institute of Marine Sciences – OKEANOS & Institute of Marine Research – IMAR, University of the Azores, Horta, Portugal; 2 University of Algarve, Campus de Gambelas, Faro, Portugal; 3 Faculty of Life and Environmental Sciences, University of Iceland, Reykjavik, Iceland; 4 Sea Mammal Research Unit, School of Biology, University of St Andrews, St Andrews, Scotland

**Keywords:** *Physeter macrocephalus*, Vertical movement, Buzzes, Foraging behaviour, Low-resolution data, Time-depth recorders

## Abstract

**Background:**

High-resolution sound and movement recording tags offer unprecedented insights into the fine-scale foraging behaviour of cetaceans, especially echolocating odontocetes, enabling the estimation of a series of foraging metrics. However, these tags are expensive, making them inaccessible to most researchers. Time-Depth Recorders (TDRs), which have been widely used to study diving and foraging behaviour of marine mammals, offer a more affordable alternative. Unfortunately, data collected by TDRs are bi-dimensional (time and depth only), so quantifying foraging effort from those data is challenging.

**Methods:**

A predictive model of the foraging effort of sperm whales (*Physeter macrocephalus*) was developed to identify prey capture attempts (PCAs) from time-depth data. Data from high-resolution acoustic and movement recording tags deployed on 12 sperm whales were downsampled to 1 Hz to match the typical TDR sampling resolution and used to predict the number of buzzes (i.e., rapid series of echolocation clicks indicative of PCAs). Generalized linear mixed models were built for dive segments of different durations (30, 60, 180 and 300 s) using multiple dive metrics as potential predictors of PCAs.

**Results:**

Average depth, variance of depth and variance of vertical velocity were the best predictors of the number of buzzes. Sensitivity analysis showed that models with segments of 180 s had the best overall predictive performance, with a good area under the curve value (0.78 ± 0.05), high sensitivity (0.93 ± 0.06) and high specificity (0.64 ± 0.14). Models using 180 s segments had a small difference between observed and predicted number of buzzes per dive, with a median of 4 buzzes, representing a difference in predicted buzzes of 30%.

**Conclusions:**

These results demonstrate that it is possible to obtain a fine-scale, accurate index of sperm whale PCAs from time-depth data alone. This work helps leveraging the potential of time-depth data for studying the foraging ecology of sperm whales and the possibility of applying this approach to a wide range of echolocating cetaceans. The development of accurate foraging indices from low-cost, easily accessible TDR data would contribute to democratize this type of research, promote long-term studies of various species in several locations, and enable analyses of historical datasets to investigate changes in cetacean foraging activity.

**Supplementary Information:**

The online version contains supplementary material available at 10.1186/s40462-023-00393-2.

## Introduction

Efficiency at foraging is crucial for predators so that enough energy is left for the remaining life-history traits, ensuring growth, reproductive success and ultimately, individual survival [1, 2]. Consequently, individual foraging success is one of the most important drivers of population dynamics [3]. However, as foraging success is difficult to estimate, long-term changes in foraging effort has been used to infer population health and the impact of natural and anthropogenic changes [4–6].

Numerous marine diving predators forage at depth challenging the direct observation of their foraging activity. The first studies of the foraging behaviour of diving predators (e.g., northern elephant seal, *Mirounga angustirostris* [7]; ringed seal, *Phoca hispida* [8]; and various cetaceans [9]) were based on occasional direct observations and the examination of stomach contents. Although the analyses of stomach contents of hunted and stranded individuals allowed to identify the main feeding habits of many marine diving predators [10], it did not enable a good understanding of their foraging effort [11].

The subsequent emergence of biologging devices (i.e., animal-attached data recorders) revolutionized the study of foraging behaviour of marine predators, allowing the continuous tracking and recording of movements and behaviour of animals at sea [12]. The first biologging devices used on marine animals measured depth as function of time [13], enabling reconstructing 2D dive profiles [14, 15]. Although these Time-Depth Recorders (TDRs) later evolved to record other parameters, here the term is used to refer to devices recording only depth over time, at a sampling rate of 1 Hz as this was the maximum accuracy provided by the available equipment. Based on the 2D movement data obtained from TDRs, numerous studies distinguished different dive phases to quantify time spent transiting, foraging and resting [16], or to identify different dive shapes to separate foraging (U-shaped) from exploratory (V-shaped) dives [17, 18].

The development of sophisticated multi-sensor tags incorporating high-resolution tri-axial accelerometers and magnetometers, depth sensors and hydrophones has enabled unprecedented views of the 3D fine-scale movement behaviour of cetaceans [19–21], especially for those species that use sound to forage. In sperm whales (*Physeter macrocephalus*), such high-resolution acoustic tags enable recording buzzes, i.e., very fast echolocation click sequences which are commonly used as indicators of prey capture attempts (PCAs) [19, 20, 22–24]. Buzzes occur most in the bottom phase of sperm whale dives and are associated with increased manoeuvring and dive inflection points [22]. Changes in movement parameters near the end of the buzzes [24] suggest an active-pursuit hunting strategy for this species [15, 25]. Greater variations in buzz rates and movement during PCAs indicated that male sperm whales also target less mobile prey and may have a more generalist diet [26].

Unfortunately, the use of multi-sensor tags has been severely constrained by their high cost, making it difficult to investigate variations in foraging effort over time or across individuals due to small sample sizes [27, 28]. The development of robust indices of sperm whale foraging activity from time-depth data would offer the possibility of using relatively inexpensive, widely available TDRs. This would not only enable increasing the sample size and duration of studies but also conducting retrospective analysis of existing low-resolution diving datasets to assess changes in foraging activity over longer time scales, a key aspect on megafauna movement ecology [28]

Earlier studies attempted to use dive metrics calculated from TDR data to estimate foraging effort and PCAs at fine-scale. For example, the number of PCAs per dive of Antarctic fur seals (*Arctocephalus gazella*) was modelled based on the vertical transit (descent and ascent rates) and recovery time at the surface [29]. For southern elephant seals (*M. leonina*) and Weddell seals (*Leptonychotes weddellii*), a broken stick algorithm was used to split 2D dives into short segments and different metrics were calculated for each dive segment [30]. Sinuosity of dive segment was found to be a good proxy of foraging behaviour in both species. Bottom time and ascent rate were the most reliable predictors of Australian fur seal (*A. pusillus doriferus*) PCAs in time-depth data [31]. While these previous studies were successful at finding reliable proxies of foraging effort in 2D dives, that could potentially be applied to several pinnipeds, there have been no attempts to develop similar indices for cetaceans.

**Fig. 1:**
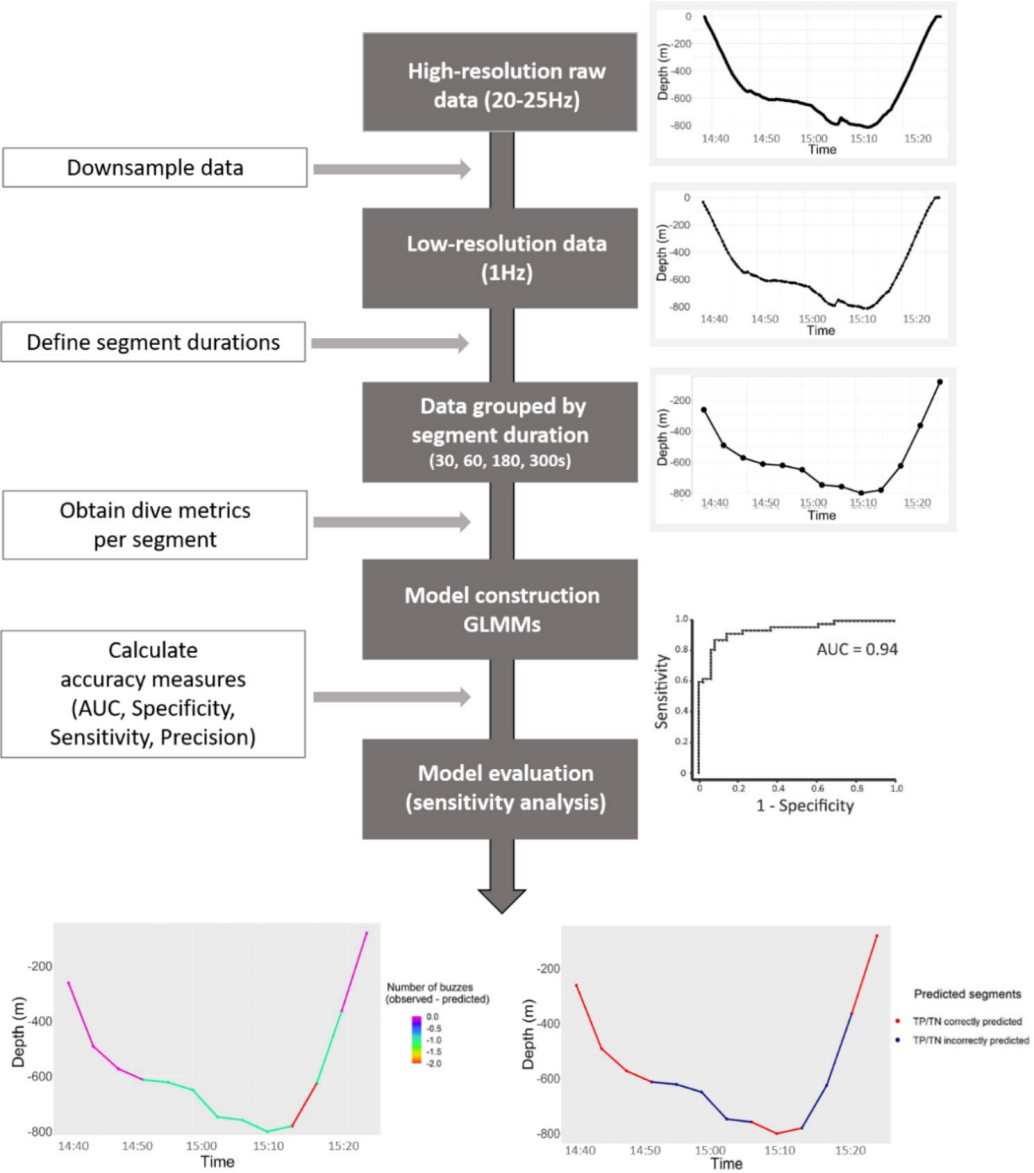
Schematic highlighting the modelling approach implemented to estimate prey capture attempts for sperm whales using low-resolution data. (1) High-resolution movement data (20-25 Hz) with identified prey captured attempts/events were downsampled to obtain low-resolution data (1 Hz). (2) The low-resolution data were grouped based on different segment durations (30, 60, 180 and 300 s). The investigated time scales of analysis (i.e. segment durations) were based on the minimum duration of prey capture attempts. (3) Dive metrics were obtained for the different segments and models (GLMMs) were constructed. (4) The predictive performance of the models was evaluated using multiple accuracy measures (AUC, Specificity, Sensitivity and Precision) and the differences between the number of prey capture attempts/events observed and predicted per segments were calculated

The objective of this study was to develop a predictive model of PCAs for sperm whales from low-resolution time-depth data. To achieve this, high-resolution movement and acoustic data from 12 sperm whales instrumented with digital acoustic recording tags (Dtags; [32, 33]) was used to extract time-depth values at a sampling frequency of 1 s and detect buzzes, considered to represent PCAs. Using this dataset, a comprehensive modelling approach (Fig. 1) was implemented by: i) analysing the dive profiles with different segment durations and calculating a suite of dive metrics for each segment duration, ii) using Generalized Linear Mixed Models (GLMMs) to identify dive metrics that best predict the number of buzzes, iii) evaluating the predictive performance of the best models using multiple accuracy measures, and finally iv) conducting a sensitivity analysis to determine whether model outputs were robust to parameter uncertainty.

## Materials and methods

### Data collection

Data were collected from 12 sperm whales instrumented with Dtags (version 3; [32, 33]) off the Azores archipelago between 2017 and 2019 (Additional file 1: Table A1). Dtags recorded 2-channel audio data (sampling frequencies of 192 kHz for 2017, and 120 kHz for 2018–2019) and collected pressure, 3-axis accelerometer, and 3-axis magnetometer data at 20 Hz in 2017, and 25 Hz in 2018–2019. The suction-cup tags were attached to the back of sperm whales using a cantilevered or hand-held pole operated from a small rigid-hulled inflatable boat (RHIB). Tags were located and recovered by radio tracking after being released from the whale. Sperm whale tagging was conducted under research permits 37/2016/DRA, 80/2017/DRA and LMAS-DRAM/2018/06 issued by the Regional Government of the Azores and followed the guidelines of the American Society of Mammalogists [34].

### Data processing

Audio data were analysed using a custom code in MATLAB (R2007b and R2016b; The Mathworks Inc., Natick, MA). The audio data were visualised using spectrograms (512 sample FFT block size, 15 s segments with 2 s overlap, Hanning window) and plotted alongside the whale’s dive profile. Clicks produced by the tagged whale were identified based on their higher received acoustic level, angle-of-arrival [33, 35] and temporal characteristics [36]. Buzz start time was defined as a change in amplitude and/or spectral content of clicks before a fast clicking rate, and buzz end time as a change in amplitude and/or spectral content or the start of a pause before the next usual click or buzz [26]. The time between the end of one buzz and the start of the following was defined as the inter-buzz interval (IBI).

Tag depth was derived from pressure readings using established methods [32]. The first dive of each individual was excluded from analysis to eliminate potential tagging effects [23]. A foraging dive was defined as being deeper than 25 m [20] and including at least one buzz [37]. Dtag depth data collected at 20 or 25 Hz were downsampled to 1 Hz to match the typical TDR sampling rate. Descent and ascent phases were determined by a continuous depth rate (depth(t_n_)-depth(t_n-1_)) higher and lower than zero, respectively. Descent and ascent phases were occasionally interrupted by brief periods in which the depth changed in the other direction. In order to define complete descent and ascent phases, those brief periods were ignored, as their short duration was negligible in relation to the dive phase duration (following the same principle in [38]). The bottom phase was defined as the period between the descent and ascent phases of the dive.

To investigate the foraging effort of sperm whales at the shortest time intervals and select the appropriate time scale of analysis, the mean buzz duration of less than 10 s reported for the species in the study area and surrounding waters was taken into account [24, 39]. Based on this buzz duration, the downsampled dataset of 1 Hz (1 s) was grouped into segments of 30, 60, 180 and 300 s, resulting in four different datasets, one for each segment duration. The package “zoo” [40] in R [41] was used to identify the start and end of each segment for each segment duration and to group the segments without overlap between them. Incomplete segments at the end of the dive profiles were excluded from the analysis (see Table A5 for the numbers of segments analysed). The dive phase assigned to the first second of each segment was used to indicate dive phase for the whole segment. In an initial exploratory analysis, the use of shorter dive segments and a broken stick algorithm to identify breakpoints in the dive profile was explored but abandoned due to poor performance.

### Dive metrics

Eighteen dive metrics were calculated for every dive segment based on the knowledge of the species’ foraging behaviour and their potential to predict buzzes [15, 19, 20, 24–26, 42] (Table 1). For each dive segment, the total number of buzzes was calculated. Sperm whales generally produce more buzzes and show increased manoeuvring, changes in body orientation and dive inflections during the bottom phase of their dives [22]. Therefore, to attempt to capture this behaviour in time-depth data, a series of parameters potentially indicative of foraging at depth (average depth, maximum depth, depth difference between the start and end of the segment), and of increased manoeuvring along the vertical axis (variance of depth) were calculated. In addition, for each segment, bottom time was calculated as the percentage of time of that segment spent at more than 60%, 70%, 80% and 90% of the dive maximum depth [43]. The production of buzzes can also be associated with strong bursts of speed and changes in acceleration [24]. Although time-depth data cannot be used to calculate forward swimming speed and acceleration, it does reflect changes in the vertical component of the animal’s motion. Vertical velocity was defined as the difference in depth (1 Hz data) between time *t*_*1*_ and time *t*_*0*_, and vertical acceleration as the difference in vertical velocity between time *t*_*1*_ and time *t*_*0*_. The average and variance of vertical velocity and acceleration were then calculated over the segment duration. Inflection points have been associated with foraging activity in sperm whales [22]. Dive inflections were defined as the moments in time *t*_*0*_ where depth was shallower or deeper than depth at *t*_*1*_ and *t*_*-1*_, and wiggles were defined as inflection points with a difference in depth > 20 m in relation to the previous inflection point [22, 42]. Steady periods (i.e., periods with equal depth) reflect the time spent at approximately the same depth, and can be indicative of prey chasing along the horizontal axis [25]. Vertical sinuosity (hereafter, sinuosity) was calculated for each dive segment, as the ratio between the vertical distance travelled in a linear path (i.e., the absolute depth difference between the start and end of the segment) and the sum of all the vertical distances the whale has actually travelled in that segment [43]. A segment with a sinuosity of 1 expresses a straight path; any deviation from a straight path decreases the sinuosity towards 0.

Prior to constructing the models, all candidate metrics were tested for collinearity using the Pearson’s correlation coefficient (Additional file 1: Table A2). Selected metrics had correlations < 0.7 and considered to be ecologically more relevant [44].

**Table 1:** Description of dive metrics calculated for each dive segment

Dive metrics	Definition	Unit
Average depth	Average depth of the segment (m)	m
Maximum depth	Maximum depth of the segment (m)	m
Variance depth	Variance in depth over the segment (m)	m
Depth difference	Difference in absolute depth between the start and end of the segment (m)	m
Time 60% max. depth	Time spent at > 60% of the maximum dive depth (seconds)	Ratio 0–1
Time 70% max. depth	Time spent at > 70% of the maximum dive depth (seconds)	Ratio 0–1
Time 80% max. depth	Time spent at > 80% of the maximum dive depth(seconds)	Ratio 0–1
Time 90% max. depth	Time spent at > 90% of the maximum dive depth(seconds)	Ratio 0–1
Vertical velocity	Difference in absolute depth between time *t*_*1*_ and time *t*_*0*_	m s-1
Average vertical velocity	Vertical velocity averaged over the segment	m s-1
Variance vertical velocity	Variance in vertical velocity over the segment	m s-1
Vertical acceleration	Absolute difference in vertical velocity between time *t*_*1*_ and time *t*_*0*_	m s-2
Average vertical acceleration	Vertical acceleration averaged over the segment	m s-2
Variance vertical acceleration	Variance in vertical acceleration over the segment	m s-2
Inflections	Point where Depth(*t*_*0*_)-Depth(t_-1_) > 0 & Depth(*t*_*1*_)-Depth(t_0_) < 0 or Depth(*t*_*0*_)-Depth(t_-1_) < 0 & Depth(*t*_*1*_)-Depth(t_0_) > 0	m
Wiggles	Inflection where depth difference to previous inflection point is > 20 m	0/1
Steady points	Point where Depth(*t*_*0*_) = Depth(t_-1_)	s
Sinuosity	Absolute depth difference / Sum vertical velocity over segment	Ratio 0–1

### Model construction and evaluation

GLMMs were used to examine the relationship between the number of buzzes and the candidate dive metrics, using a separate model for each dive segment duration. Models were fitted with a Poisson distribution and included individual whale as a random effect (R package “lme4”; [45]). A cross-validation procedure was applied by partitioning the data into a training dataset containing 67% of the data (8 of the 12 individuals), used to calibrate the models, and a test dataset with the remaining 33% of the data (4 individuals) to evaluate the models. The GLMMs were built using a backward selection of the variables, and the best model was chosen based on the lowest Akaike’s Information Criterion (AIC) [46]. Marginal and conditional R^2^ values describing the variance explained by the models [47] were calculated with the R package “MuMIn” [48].

Model predictive performance was evaluated using multiple accuracy measures ([49, 50]; Additional file 1: Table A3): AUC - the area under the ROC (receiver operating characteristic) curve, Sensitivity - the proportion of presences correctly predicted, Specificity - the proportion of absences correctly predicted, and Precision - the proportion of true positives to total predicted positives [51], which were calculated by generating a ROC curve using R package “ROCR” [52]. The AUC index ranges from 0 to 1; AUC ≤ 0.6 indicate a discrimination ability no better than random, 0.6–0.7 indicate moderate predictive performance, 0.7–0.8 as good, 0.8–0.9 as very good and > 0.9 as excellent [53]. Finally, the absolute difference between the total number of observed and predicted buzzes per segment and per dive were calculated for each individual whale.

A sensitivity analysis was performed separately for each of the four segment durations to determine whether model outputs were robust to the different dive metrics values obtained from the 12 individuals. The analysis was conducted by randomly selecting a training dataset (8 of the 12 individuals) to obtain the dive metrics’ estimates of the best-fitting model and evaluate them on the test dataset (remaining 4 of the 12 individuals) through assessing their significance value, repeating this procedure 100 times. All accuracy measures (sensitivity, specificity, precision and AUC) were calculated for each model run, and the median and standard deviation of each measure were obtained from the 100 runs. Lastly, in order to assess the effect of the type of data included in the models, models with the same segment durations were built using only data from the bottom phase of dives and the resulting accuracy measures were compared to the measures obtained from the models including all dive data (descent, bottom and ascent phases).

## Results

The dataset from the 12 sperm whales included 103 foraging dives and 1278 buzzes, with a mean of 9 (SD: 7) foraging dives per individual and 12 (SD:6) buzzes per dive (Additional file 1: Table A1). The number of segments for all whales combined ranged from 915 (300 s) to 9068 segments (30 s), with the largest number of segments allocated to the bottom dive phase (mean: 62% of the segments; SD:2) followed by the ascent (mean: 21%; SD: 2) and descent dive phase (mean: 17%; SD: 2) (Additional file 1: Tables A4 & A5). Only 12% of the 30 s segments contained at least one buzz, whereas this percentage was considerably higher (58%) for 300 s segments. The number of buzzes per segment ranged from 0 to 2 for 30 s segments, and 0 to 7 for 300 s segments (Additional file 1: Table A4). For all segment durations analysed, most dive segments were at 500–900 m depth, where the highest number of buzzes occurred (Additional file 1: Fig. A1), with only a few segments deeper than 1000 m.

### Relationship between buzz production and diving behaviour

**Fig. 2:**
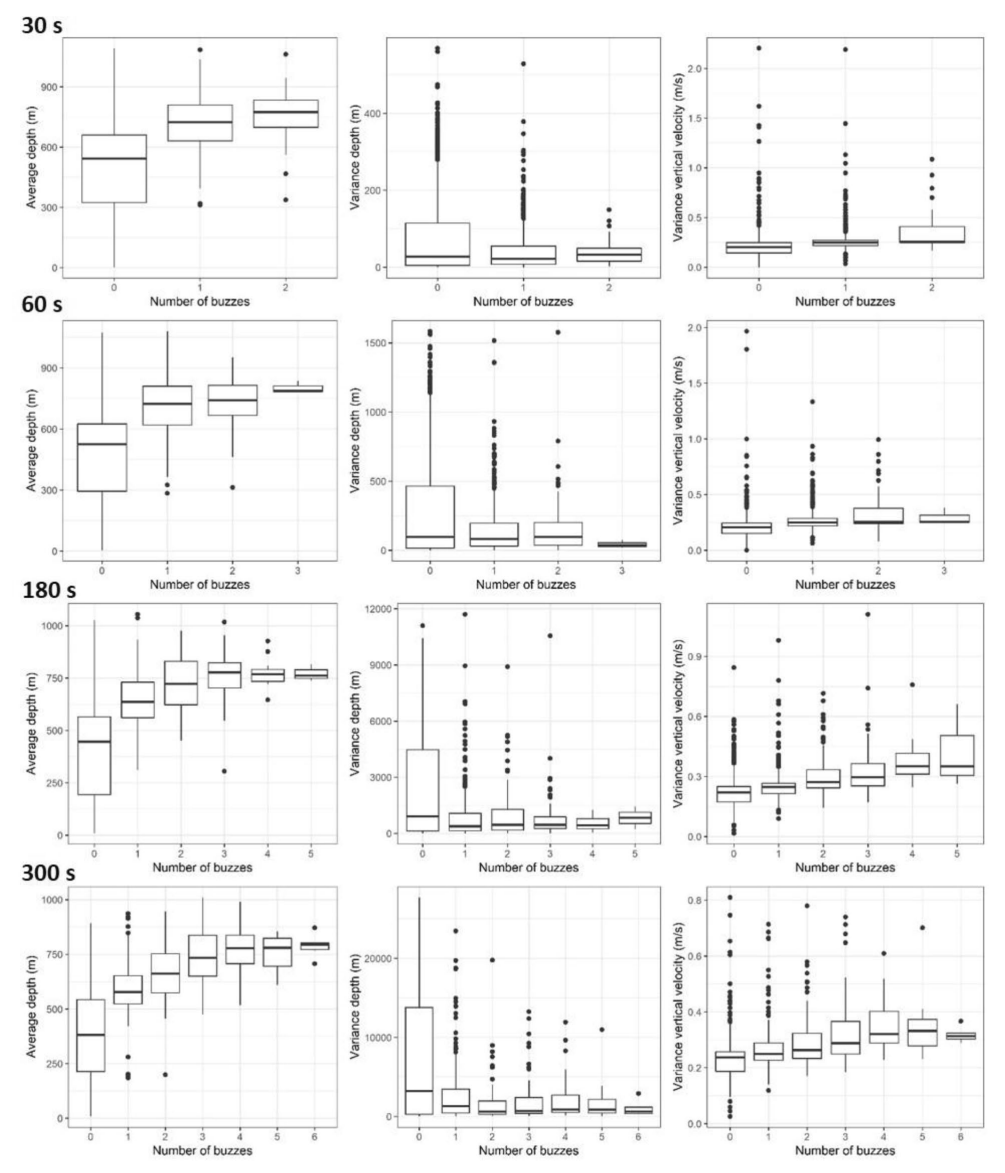
Distribution of dive metrics per number of buzzes for segments of 30, 60, 180 and 300 s. The horizontal line represents the median, the box represents the 25th and 75th percentiles, the whiskers represent the extreme values within 1.5 times the length of the box, and dots beyond the end of the whiskers are outlier points [54]

The best-fitting GLMM for each segment duration (30, 60, 180 or 300 s) analysed included average depth, variance of depth, and variance of vertical velocity. The number of buzzes showed a positive linear relationship with the average depth and variance of vertical velocity, and a negative relationship with the variance of depth (Table 2; Fig. 2). The three variables retained in the best-fitting models were significant in all 100 models ran with different combinations of individuals, showing the robustness of these metrics to multiple simulations (Table 2). The average depth and variance of vertical velocity were also significant when models were built using only data from the bottom phase of dives (Additional file 1: Table A7), and showed the same relationship with the number of buzzes as with all dive data (Additional file 1: Fig. A2). However, the variance of depth was less important in those models. Models for segments of 180 s and 300 s had a higher variance explained (marginal and conditional R^2^ values ≥ 0.45) than segments with 30 s and 60 s (< 0.25) (Table 2).

**Table 2:** Summary of the modelling outputs for dive segments of 30, 60, 180 and 300 s. Models were run 100 times, and the significance of explanatory dive metrics was evaluated with p values < 0.01. Median and standard deviation (shown in brackets) values for marginal and conditional R^2^ were obtained from the sensitivity analysis with 100 model runs

Segment duration	Dive metric	Estimate	Std. Error	Z value	*n*-significant (*n*/100)	R^2^ marginal	R^2^ conditional
30 s	Average depth	1.14014	0.06897	16.53	100/100	0.13 (0.03)	0.13 (0.03)
	Variance depth	-0.14611	0.04566	-3.2	98/100		
	Variance vertical velocity	0.23185	0.01722	13.46	100/100		
60 s	Average depth	0.99658	0.07358	13.544	100/100	0.22 (0.04)	0.24 (0.04)
	Variance depth	-0.35951	0.06249	-5.753	100/100		
	Variance vertical velocity	0.23359	0.02049	11.399	100/100		
180s	Average depth	0.95534	0.06617	14.437	100/100	0.46 (0.05)	0.49 (0.05)
	Variance depth	-0.35571	0.06988	-5.09	100/100		
	Variance vertical velocity	0.18681	0.02726	6.854	100/100		
300s	Average depth	0.57066	0.04762	11.984	100/100	0.54 (0.04)	0.58 (0.04)
	Variance depth	-0.46369	0.06395	-7.25	100/100		
	Variance vertical velocity	0.21382	0.02832	7.551	100/100		

### Predictive ability of the models

The best model for the 30 s and 60 s dive segments had a median AUC ≤ 0.62, indicating a low to moderate predictive performance (Table 3). These models had an excellent specificity (> 90% of segments without buzzes were correctly classified) and a low sensitivity (< 30% of segments with buzzes were correctly classified). The best models for 180 s and 300 s had a good predictive performance (median AUC ≥ 0.77), and an excellent sensitivity (> 90% of segments with buzzes correctly classified). The model for the 180 s segments had a higher specificity (64%) than the model based on 300 s segments (56%). Segments with 180 s and 300 s resulted in a higher precision (≥ 70%) than segments with 30 s and 60 s (< 60%). Similar results were obtained on all accuracy measures when using only data from the bottom phase, except a much lower predictive performance (AUC) and specificity for segments with 180 s and 300 s (Table 3).

**Table 3:** Results of the accuracy measures (AUC, sensitivity, specificity and precision) obtained from the sensitivity analysis for segments of 30, 60, 180 and 300 s. Median and standard deviation (shown in brackets) values were obtained from the sensitivity analysis with 100 model runs

Data type	Segment duration	AUC	Sensitivity	Specificity	Precision
All dive data	30 s	0.52 (0.00)	0.05 (0.02)	0.99 (0.01)	0.49 (0.13)
	60 s	0.62 (0.04)	0.29 (0.10)	0.94 (0.02)	0.59 (0.09)
	180 s	0.78 (0.05)	0.93 (0.06)	0.64 (0.14)	0.70 (0.12)
	300 s	0.77 (0.04)	0.96 (0.02)	0.56 (0.08)	0.77 (0.06)
Bottom phase data	30 s	0.51 (0.01)	0.04 (0.01)	0.99 (0.01)	0.56 (0.14)
	60 s	0.60 (0.04)	0.29 (0.13)	0.93 (0.06)	0.67 (0.12)
	180 s	0.50 (0.02)	1.00 (0.01)	0.00 (0.05)	0.68 (0.10)
	300 s	0.50 (0.00)	1.00 (0.00)	0.00 (0.01)	0.82 (0.07)

Models for segments of 30 s and 60 s correctly identified the number of buzzes in 87% and 77% of the segments, respectively (Additional file 1: Table A6). When overestimating or underestimating the number of buzzes, their bias usually ranged from 1 to -1 buzz per segment, with less than 0.20% of the segments with an absolute bias of greater than 3 buzzes. Although there were small differences between the total number of buzzes observed and predicted per segment, the large number of segments within a dive (for 30s a median of 105 segments (SD: 94.41); for 60s a median of 57 segments (SD: 49.20)) led to large absolute differences in the numbers of buzzes per dive (Table A4). A median difference of 9 and 6 buzzes was obtained from models of 30 s and 60 s, respectively, nearly doubling the median number of buzzes per dive (median of 10 buzzes), and resulting on an absolute difference in predicted buzzes of more than 70% (Table 4). On the other hand, models of 180 s predicted the exact number of buzzes in 54% of segments, overestimated the number of buzzes in 23% of segments, and underestimated in 23%, with most values within the − 2 to + 2 range (Additional file 1: Table A6). Models of 300 s had the lowest percentage of accurate identifications of the number of buzzes (accurately predicted in 44% of the segments; Additional file 1: Table A6). Models of 180 s and 300 s segments yielded the smallest difference between observed and predicted number of buzzes per dive, with a median absolute difference of 4 and 3 buzzes per dive, respectively, representing an absolute difference in predicted buzzes of 25–30% (Table 4).

**Table 4:** Difference between the total number of observed and predicted buzzes per dive for each segment duration, in terms of absolute value and percentage of segments Median and standard deviation (shown in brackets) values were obtained from the sensitivity analysis with 100 model runs

Data type	Segment duration	Absolute difference nº buzzes/dive |observed – predicted|	% Absolute difference |observed – predicted|	Nº observed buzzes per dive	Nº segments per dive
All dive data	30 s	9 (7.88)	100 (46.05)	10 (7.41)	105 (94.41)
	60 s	6 (7.97)	73 (57.96)	10 (7.38)	57 (49.20)
	180 s	4 (4.76)	30 (96.05)	10 (7.35)	17 (16.31)
	300 s	3 (4.71)	25 (82.41)	11 (7.32)	12 (9.17)
Bottom phase data	30 s	9 (5.55)	100 (43.74)	10 (5.64)	70 (56.34)
	60 s	7 (5.08)	83 (66.85)	10 (5.58)	36 (28.68)
	180 s	4 (3.33)	37 (112.90)	11 (5.86)	12 (10.81)
	300 s	4 (3.46)	36 (127.78)	11 (5.68)	8 (6.35)

**Fig. 3:**
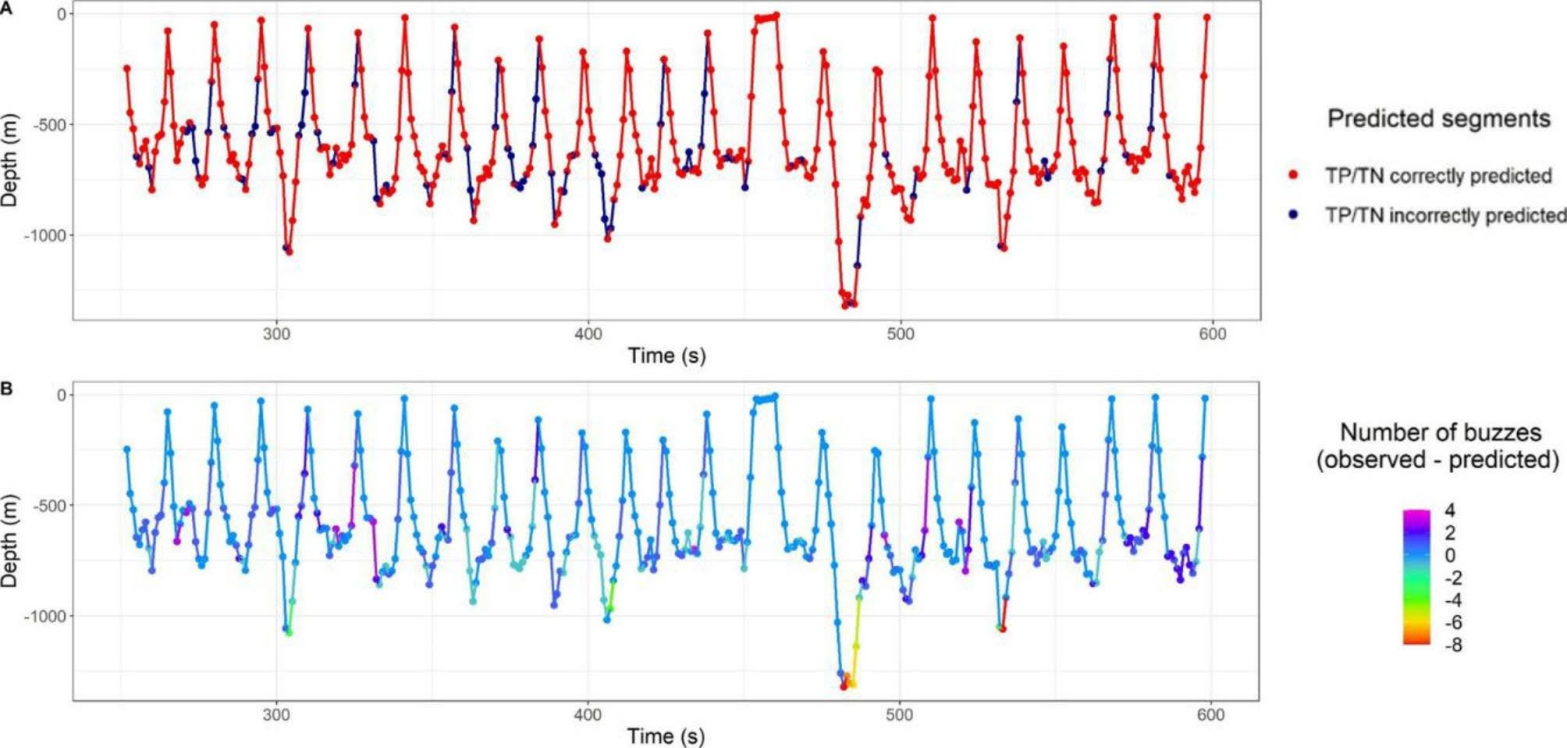
** A)** Segments with their number of buzzes correctly or incorrectly predicted by the model for the dive profile of whale sw19_088a and segment duration of 180 s. **B)** Difference in the number of buzzes observed and predicted by this model

Differences between the observed and predicted buzzes occurred in all dive phases but most incorrect predictions were found in the bottom phase for all segment durations (Fig. 3, Additional file 1: Figs. A3 & A4; Table A8). The highest overestimations of the number of buzzes were found at the deepest part of the dives (> 1000 m), mainly for models of 180 and 300 s (Additional file 1: Fig. A5).

## Discussion

In this study, a model was developed to detect the number of buzzes within sperm whale dives, when no concurrent acoustic information was available and solely based on dive metrics derived from 1 Hz time-depth profiles. Other studies developed similar methods to predict foraging activity of pinnipeds from time-depth data [30, 43]. However, this is the first approach capable of predicting prey capture attempts (PCAs) by sperm whales at the scale of a few minutes, along the entire dive. The sensitivity analysis showed that the model was able to detect the presence and number of buzzes within dive segments from different individuals with good accuracy. Overall, models for segments of 180 s showed the highest accuracy scores, with a good predictive performance, an excellent identification of segments with buzzes, and good identification of segments without buzzes, as well as a small difference between the total number of buzzes observed and predicted per dive (Table 3; Additional file 1: Table A6). Models including all dive data performed better than those including only data from the bottom phase (Table 3). This model, therefore, constitutes a robust tool to estimate sperm whale foraging effort at fine spatial and temporal scales, representing a significant improvement over previous approaches using surface time [55], or dive duration, surface interval and distance travelled during a dive cycle [56].

### Dive metrics used to detect buzzes

In the Azores archipelago, sperm whales forage mainly between 700 and 1200 m depth [39]. The fact that foraging activity takes place within such a specific range explains why average depth was an important predictor in the model, contributing to the model’s ability to discriminate between segments with and without buzzes (Fig. 2; Table 2). In addition, sperm whales produce more buzzes during the bottom phase of the dives [39], as in other regions [19]. Two of the metrics used to measure time spent in the bottom phase (time at 60% and 70% of the maximum depth) were correlated to average depth, and the other two metrics (time at 80% and 90%) were not retained in the best-fitting models (Table 2). Although the number of buzzes had a positive linear relationship with average depth for segments < 800 m depth, this relationship was constant for segments deeper than 800 m, resulting in small differences on average depth for segments with 3–5 buzzes. This partly explains why differences between observed and predicted buzzes increased with depth. The dataset used to develop the models included few dives > 1000 m and model accuracy in the deepest segments was substantially reduced (Additional file 1: Figs. A1 & A5).

The presence of buzzes was associated with reduced depth variance, most likely because most segments without buzzes occurred during the descent and ascent phases of a dive (Fig. 2). Average vertical velocity (± SD) during the descent and ascent phases of dives for sperm whales in the Azores was 1.35 ± 0.21 m s^-1^ and 1.60 ± 0.19 m s^-1^, respectively [33, 39]. This means that changes in depth between the start and end of the longest segments can surpass 200 m, so the variance in depth within these segments is high. Conversely, the results showed that variance in depth over time scales of a few minutes during the bottom phase of the dive, where most buzzes were produced, was substantially smaller. Although variance in depth was a good predictor of the presence and absence of buzzes, it changed little with the number of buzzes, suggesting that consecutive buzzes within a segment concentrate in a restricted depth range.

The production of buzzes by sperm whale has been linked to strong bursts of speed [15, 24]. To capture the vertical component of these bursts, the variance in vertical velocity and in vertical acceleration were calculated for each dive segments but only the former had a significant effect on the number of buzzes per segment (Fig. 2). A higher number of buzzes occurred in segments with higher variance in vertical velocity, possibly reflecting sudden accelerations during active prey chases, followed by slowdowns after prey capture [24].

Vertical sinuosity in diving data, quantified by inflection points and wiggles, has been related to successful prey capture in baleen whales and deep diving predators [57–59]. Even though one could expect wiggles and inflection points to be good predictors of the number of buzzes, that was not the case in this study. It is possible that wiggles and inflection points did not capture the associated three-dimensional movements associated with buzz production [24].

### Model predictive performance

The models for segments of 180 and 300 s showed a very good predictive performance, considerably higher than models for 30 and 60 s segments (Table 3). Although models for 30 and 60 s had a slightly higher specificity (proportion of true negative correctly predicted) than models for 180 and 300 s segments, their sensitivity (proportion of true positive correctly predicted) was nearly four times lower. The 300 s model had worse results than the 180 s model at predicting the exact number of buzzes per segment (54% and 44% of the segments for 180 and 300 s, respectively). Additionally, the 180 s model correctly detected the presence or absence of buzzes in 78% of new observations (test datasets). Thus, 180 s was considered the most suitable time scale for modelling the number of buzzes.

The performance of this model was similar to that of models developed to predict PCAs in 2D time-depth data of southern elephant seals and Weddell seals [29, 30, 43]. Unlike these models that predicted PCAs at the dive scale [30, 43] or, at best, at 30 minutes and hourly scales [29], the model developed in this study predicted PCAs at much higher resolution (3 minutes). In addition, when comparing the overall performance of this model with an automated method to detect PCAs in high-resolution data from Risso’s dolphins (true positive rate of 0.41; [60]), the approach developed on the present study based on low-resolution data performed much better (true-positive rate of 0.92) (Table 3).

Hence, the model showed a great potential to estimate PCAs from low-resolution time-depth data for sperm whales. Nonetheless, there was still space for improvement. For instance, there was considerable overlap in the distribution of all dive variables retained in the final models across different numbers of buzzes (Fig. 2). This was especially evident for the variance in depth and for the average depth in segments with ≥ 3 buzzes, and largely explains why model performance at predicting presence/absence of buzzes was substantially better than at predicting the exact number of buzzes. The positive linear relationship between average depth and number of buzzes (Table 2), influenced by the reduced number of segments with buzzes at > 1000 m, may also explain the overestimation of buzzes in the deepest dive segments (Additional File 1; Fig. A5). Lastly, movement signatures of foraging sperm whales are likely to vary with prey species, which could affect the accuracy of this model. However, it is not possible to know how much of the lack of accuracy of the model is due to sperm whales targeting different prey species, as previous studies have not been able to collect these data on sperm whales. The integration of video recorders on high-resolution tags could allow discriminating prey species and help refining this model [61].

### Foraging ecology applications

Information on feeding events is critical to have a better understanding of the foraging activity of marine top predators. Low-resolution data offer a simplified representation of the complex diving behaviour revealed by high-resolution movement tags [59, 62–64]. Here a model was developed, for the first time, that predicts the number of PCAs, and consequently foraging effort, from low-resolution time-depth data for sperm whales. The model showed good predictive performance at relatively short-time scales (180 seconds) for whales tagged in different years, and therefore has great potential to investigate the fine-scale foraging activity of sperm whales within the Azores archipelago, help identify areas or times where individuals maximize foraging effort around the study area, and study the factors driving foraging behaviour and habitat preferences [65, 66]. Nonetheless, to investigate if this model is transferrable to other locations it would be necessary to validate that the selected dive metrics have a good predictive capacity to identify PCAs of sperm whales from other geographic areas and populations segments, such as males in northern latitudes, which have been shown to forage at different depths [20] than sperm whales in the Azores [33]). Additionally, the same approach could potentially be applied to other odontocetes species known to produce buzzes [67, 68], enabling more accurate estimations of foraging effort than the coarse foraging indexes typically derived from time-depth profiles. However, this would likely require one to adapt the time scale of the segments to the buzz duration of the target species.

The modelling approach implemented on this study might also be applied successfully to time-depth datasets from other marine megafauna species [69, 70]. For this, it is recommended to perform the following five steps (Fig. 1): 1) Acquire high-resolution movement data with identified prey capture attempts/events; 2) Downsample the high-resolution data to match the resolution of the time-depth data (typically 1 Hz); 3) Define the appropriate time scale of analysis (segments durations) based on the duration of the prey capture attempts/events, and grouped the low-resolution data based on this duration on different segment durations; 4) Choose the dive metrics based on the knowledge of the species’ foraging behaviour and their potential to predict prey capture attempts/events, and construct the models (i.e., GLMMs) using the dive metrics obtained for the different segment durations; and 5) Evaluate the predictive performance of the models using multiple accuracy measures.

The implementation of the modelling approach of this study with sperm whales or other marine megafauna species could contribute to maximize the potential application of already existing and future datasets obtained from low-cost and widely available TDRs. Improved predictions of foraging events in these data would enable the re-interpretation of extensive datasets available, and provide much needed information on the foraging behaviour of marine megafauna species [28]. Furthermore, this approach could help to increase the sample sizes required for population-level inference in movement ecology studies and overcome the limiting factor of the cost of the multi-sensor tags [71]. In addition, the results obtained in this study are a critical step towards the development of a simple automated PCA detector that, in the future, could be integrated onboard satellite-linked tags [72], providing summarised information on foraging effort over long time-scales. Such developments would be important to study, for example, the energetic consequences of migration and long-distance movements of marine megafauna species. Finally, analysing time-series datasets of diving activity would allow investigating potential changes in the foraging behaviour of these species in response to climate or other anthropogenic changes [5]

## Electronic supplementary material

Below is the link to the electronic supplementary material.

Additional file 1: Contains supplementary tables and figures. The file contains a summary of tag data for 12 sperm whales tagged off the Azores in 2017–2019 (Table A1), a Pearson’s correlations diagram of all dive metrics (Table A2), a table with the accuracy measures used in model evaluation (Table A3), a table with the number of buzzes and percentage of segments with and without buzzes per segment duration (Table A4), a table with the number of segments per segment duration, individual and dive phase (Table A5), a table with the difference between the total number of observed and predicted buzzes per segment for each segment duration (Table A6), a summary of modelling outputs for models built only with data from the bottom phase of the dives for each segment duration (Table A7), a table with absolute differences between the total number of observed and predicted buzzes per dive for each segment duration (Table A8), a figure with number of buzzes observed by depth bins and percentage of the number of segments per depth bin for each segment duration (Fig. A1), the distribution of dive metrics per number of buzzes for each segment duration using only data from the bottom dive phase (Fig. A2), a figure with the differences between the total number of observed and predicted buzzes per segment at different dive phases for each segment duration shown as a percentage of the segments (Fig. A3), a figure with the differences between the total number of observed and predicted buzzes per segment at different dive phases for each segment duration shown as the total number of segments (Fig. A4), a figure with the differences between the total number of observed and predicted buzzes per segment at each depth for each segment duration (Fig. A5).

## Data Availability

The datasets used and/or analysed during the current study are available from the corresponding author on reasonable request.
