# Supplementary material for: Predictive model of sperm whale prey capture attempts from time-depth data
Source: Mov Ecol. 2023 Jun 8;11:33. doi: 10.1186/s40462-023-00393-2 (PMC10251647; doi:10.1186/s40462-023-00393-2)
Supplement: Supplementary file 1 — Additional file 1: Contains supplementary tables and figures. The file contains a summary of tag data for 12 sperm whales tagged off the Azores in 2017–2019 (Table A1), a Pearson’s correlations diagram of all dive metrics (Table A2), a table with the accuracy measures used in model evaluation (Table A3), a table with the number of buzzes and percentage of segments with and without buzzes per segment duration (Table A4), a table with the number of segments per segment duration, individual and dive phase (Table A5), a table with the difference between the total number of observed and predicted buzzes per segment for each segment duration (Table A6), a summary of modelling outputs for models built only with data from the bottom phase of the dives for each segment duration (Table A7), a table with absolute differences between the total number of observed and predicted buzzes per dive for each segment duration (Table A8), a figure with number of buzzes observed by depth bins and percentage of the number of segments per depth bin for each segment duration (Fig. A1), the distribution of dive metrics per number of buzzes for each segment duration using only data from the bottom dive phase (Fig. A2), a figure with the differences between the total number of observed and predicted buzzes per segment at different dive phases for each segment duration shown as a percentage of the segments (Fig. A3), a figure with the differences between the total number of observed and predicted buzzes per segment at different dive phases for each segment duration shown as the total number of segments (Fig. A4), a figure with the differences between the total number of observed and predicted buzzes per segment at each depth for each segment duration (Fig. A5). [file 40462_2023_393_MOESM1_ESM.docx]

**Supplementary information:**

**Additional file 1**

**Predictive model of sperm whale prey capture attempts from time-depth data**

Sergi Pérez-Jorge^1*^, Cláudia Oliveira^1*^, Esteban Iglesias Rivas^2^, Rui Prieto^1^, Irma Cascão^1^, Paul Wensveen^3^, Patrick J. O. Miller^4^ and Mónica A. Silva^1^

^1^ Institute of Marine Sciences – OKEANOS & Institute of Marine Research – IMAR, University of the Azores, Horta, Portugal

^2^ University of Algarve, Campus de Gambelas, Faro, Portugal

^3^ Faculty of Life and Environmental Sciences, University of Iceland, Reykjavik, Iceland

^4^ Sea Mammal Research Unit, School of Biology, University of St Andrews, Scotland

*Corresponding authors (shared first authorship):

[sergiperezjorge@gmail.com](mailto:sergiperezjorge@gmail.com)

**Table A1.** Summary of tag data for 12 sperm whales tagged off the Azores in 2017-2019.

| Whale ID | Tagging date | Duration of recordings (hh:mm) | Nº foraging dives | Nº buzzes | Mean nº buzzes per foraging dive (SD) |
| --- | --- | --- | --- | --- | --- |
| sw17_194a | 13-07-2017 | 08:57 | 8 | 135 | 17 (4) |
| sw18_170a | 19-06-2018 | 04:22 | 3 | 29 | 10 (4) |
| sw18_172a | 21-06-2018 | 05:04 | 5 | 94 | 19 (4) |
| sw18_177a | 26-06-2018 | 06:21 | 4 | 87 | 22 (2) |
| sw19_137a | 17-05-2019 | 09:28 | 8 | 120 | 15 (5) |
| sw19_158a | 07-06-2019 | 07:51 | 6 | 67 | 11 (7) |
| sw19_160a | 09-06-2019 | 25:45 | 22 | 180 | 8 (5) |
| sw19_163a | 12-06-2019 | 13:54 | 11 | 85 | 8 (2) |
| sw17_203a | 22-06-2017 | 17:13 | 7 | 78 | 11 (1) |
| sw18_173a | 22-06-2018 | 03:13 | 2 | 9 | 5 (1) |
| sw18_292a | 19-10-2018 | 06:35 | 4 | 49 | 12 (4) |
| sw19_088a | 29-03-2019 | 23:17 | 23 | 345 | 15 (5) |
| Total |  | **132:00** | **103** | **1278** | **12 (6)** |

**Table A2.** Pearson’s correlations diagram of all dive metrics.

**Table A3.** Accuracy measures used in model evaluation. The index *s* is the segment number, TP = true positive, TN = true negative, FP = false positive and FN = false negative. The AUC was calculated from the ROC curve using the trapezoid method.

| **Accuracy measure** | | **Measure description** | **Equation** |
| --- | --- | --- | --- |
| Specificity | | Proportion of true negatives correctly predicted | $\frac{{TN}_{S}}{({FP}_{S}+{TN}_{S})}$ |
| Sensitivity | | Proportion of true positives correctly predicted | $\frac{{TP}_{S}}{({TP}_{S}+{FN}_{S})}$ |
| Precision | Proportion of true positives to total predicted positives | | $\frac{{TP}_{S}}{({TP}_{S}+{FP}_{S})}$ |
| AUC | | Area below the Receiver Operating Characteristics (ROC) curve; an overall measure of classification performance | -Plot between Sensitivity on the Y-axis and (1-Specificity) on the X-axis |

**Table A4.** Number of segments with 0-7 buzzes per segment duration, and percentage of segments with and without buzzes per segment duration.

| **Nº of buzzes** | **Nº of segments per segment duration** | | | |
| --- | --- | --- | --- | --- |
|  | **30 s** | **60 s** | **180 s** | **300 s** |
| 0 | 7844 | 3494 | 771 | 338 |
| 1 | 1171 | 1004 | 388 | 217 |
| 2 | 53 | 128 | 242 | 144 |
| 3 | 0 | 6 | 98 | 129 |
| 4 | 0 | 0 | 23 | 60 |
| 5 | 0 | 0 | 4 | 17 |
| 6 | 0 | 0 | 0 | 9 |
| 7 | 0 | 0 | 0 | 1 |
| **Total** | **9068** | **4632** | **1526** | **915** |
|  |  |  |  |  |
| **with buzzes (presences)** | 13% | 25% | 49% | 63% |
| **without buzzes (absences)** | 87% | 75% | 51% | 37% |

**Table A5.** Number of segments per segment duration, individual and dive phase.

**Table A6.** Difference between the total number of observed and predicted buzzes per segment for each segment duration, summarized in terms of the total number of segments, the percentage (%) of the segments, and the total difference in the number of buzzes. Results obtained from the sensitivity analysis with 100 model runs.

**Table A7.** Summary of modelling outputs for models built only with data from the bottom phase of the dives for each segment duration. Models were run 100 times, and the significance of explanatory dive metrics was evaluated with p values <0.01. Median and standard deviation (shown in brackets) values for marginal and conditional R^2^ were obtained from the sensitivity analysis with 100 model runs.

| **Segment duration** | **Dive metric** | **Estimate** | **Std. Error** | **Z value** | ***n*-significant (*n*/100)** | **R^2^ marginal** | **R^2^ conditional** |
| --- | --- | --- | --- | --- | --- | --- | --- |
| 30 s | Average depth | 0.144482 | 0.044559 | 3.242 | 96/100 |  |  |
|  | Variance depth | -0.004757 | 0.035213 | -0.135 | 1/100 | 0.02 (0.02) | 0.04 (0.01) |
|  | Variance vertical velocity | 0.223787 | 0.017427 | 12.841 | 100/100 |  |  |
| 60 s | Average depth | 0.1801 | 0.03656 | 4.925 | 98/100 |  |  |
|  | Variance depth | -0.06352 | 0.03314 | -1.917 | 1/100 | 0.03 (0.02) | 0.07 (0.02) |
|  | Variance vertical velocity | 0.20962 | 0.01951 | 10.745 | 100/100 |  |  |
| 180 s | Average depth | 0.26069 | 0.05447 | 4.786 | 97/100 |  |  |
|  | Variance depth | -0.12398 | 0.04729 | -2.622 | 9/100 | 0.06 (0.05) | 0.18 (0.04) |
|  | Variance vertical velocity | 0.18693 | 0.03557 | 5.255 | 100/100 |  |  |
| 300 s | Average depth | 0.33603 | 0.05955 | 5.643 | 97/100 |  |  |
|  | Variance depth | -0.03414 | 0.0424 | -0.805 | 28/100 | 0.10 (0.05) | 0.24 (0.04) |
|  | Variance vertical velocity | 0.09952 | 0.03684 | 2.702 | 73/100 |  |  |

**Table A8.** Absolute difference between the total number of observed and predicted buzzes per dive for each segment duration. Median and standard deviation (shown in brackets) values were obtained from the sensitivity analysis with 100 model runs.

| **Segment duration** | **Dive phase** | | | **Absolute difference nº buzzes/dive**  **\|observed – predicted\|** | | |  |
| --- | --- | --- | --- | --- | --- | --- | --- |
| 30 s | Descent | | | 1 (1.97) | | |  |
|  | Bottom | | | 9 (7.57) | | |  |
|  | Ascent | | | 1 (1.14) | | |  |
| 60 s | Descent | | | 0 (1.78) | | |  |
|  | Bottom | | | 7 (6.61) | | |  |
|  | Ascent | | | 1 (1.02) | | |  |
| 180 s | Descent | | | 0 (0.77) | | |  |
|  | Bottom | | | 4 (5.16) | | |  |
|  | Ascent | | | 0 (0.69) | | |  |
| 300 s | Descent | | | 0 (0.70) | | |  |
|  | Bottom | | | 3 (4.43) | | |  |
|  | Ascent | | | 0 (1.18) | | |  |
|  | |  |  | |  |  | |


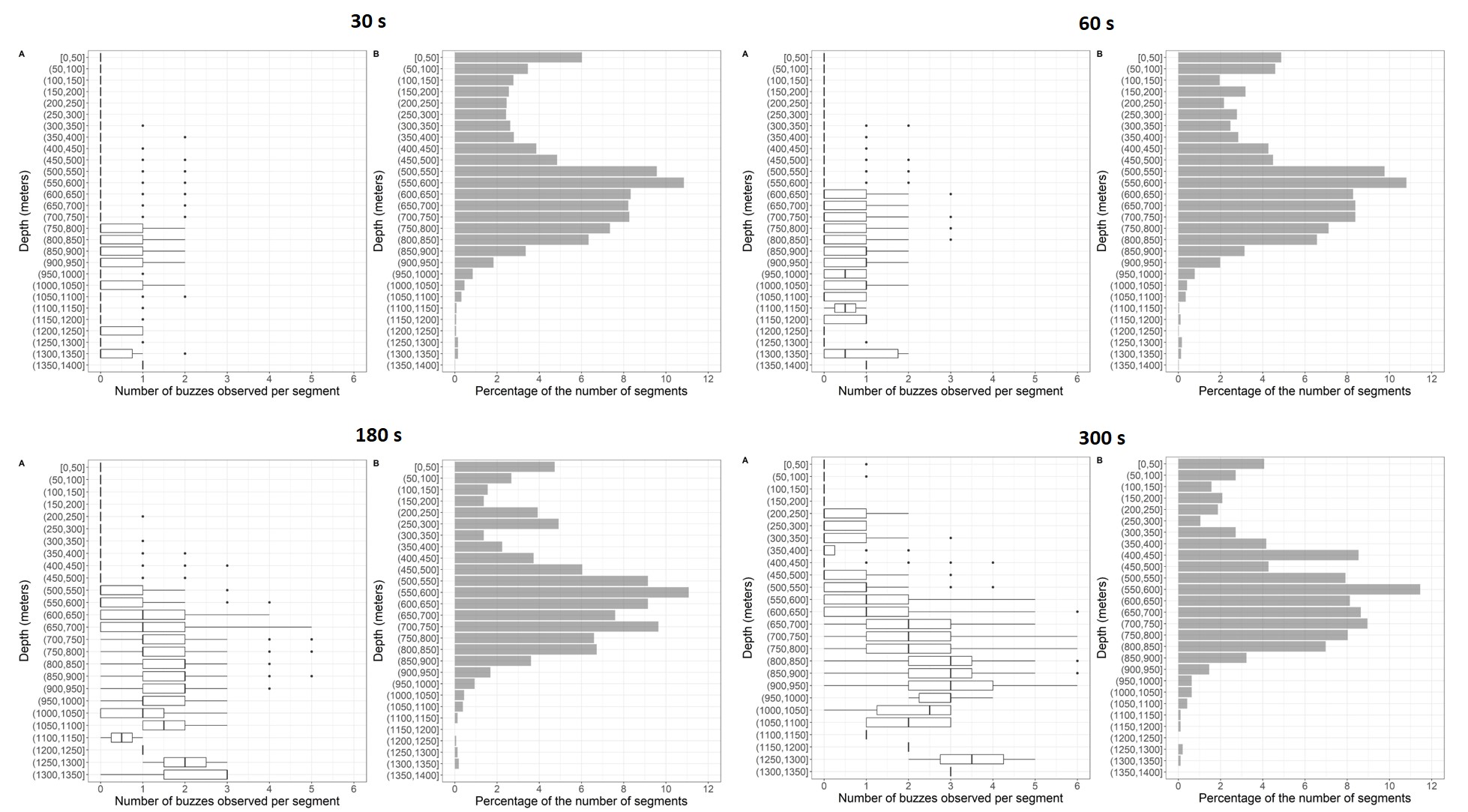


**Fig. A1. A)** Number of buzzes observed by depth bins for each segment duration. The vertical dark line represents the median, the box represents the 25^th^ and 75^th^ percentiles and the whiskers represent the extreme values within 1.5 times the length of the box. **B)** Percentage of the number of segments per depth bin.

**
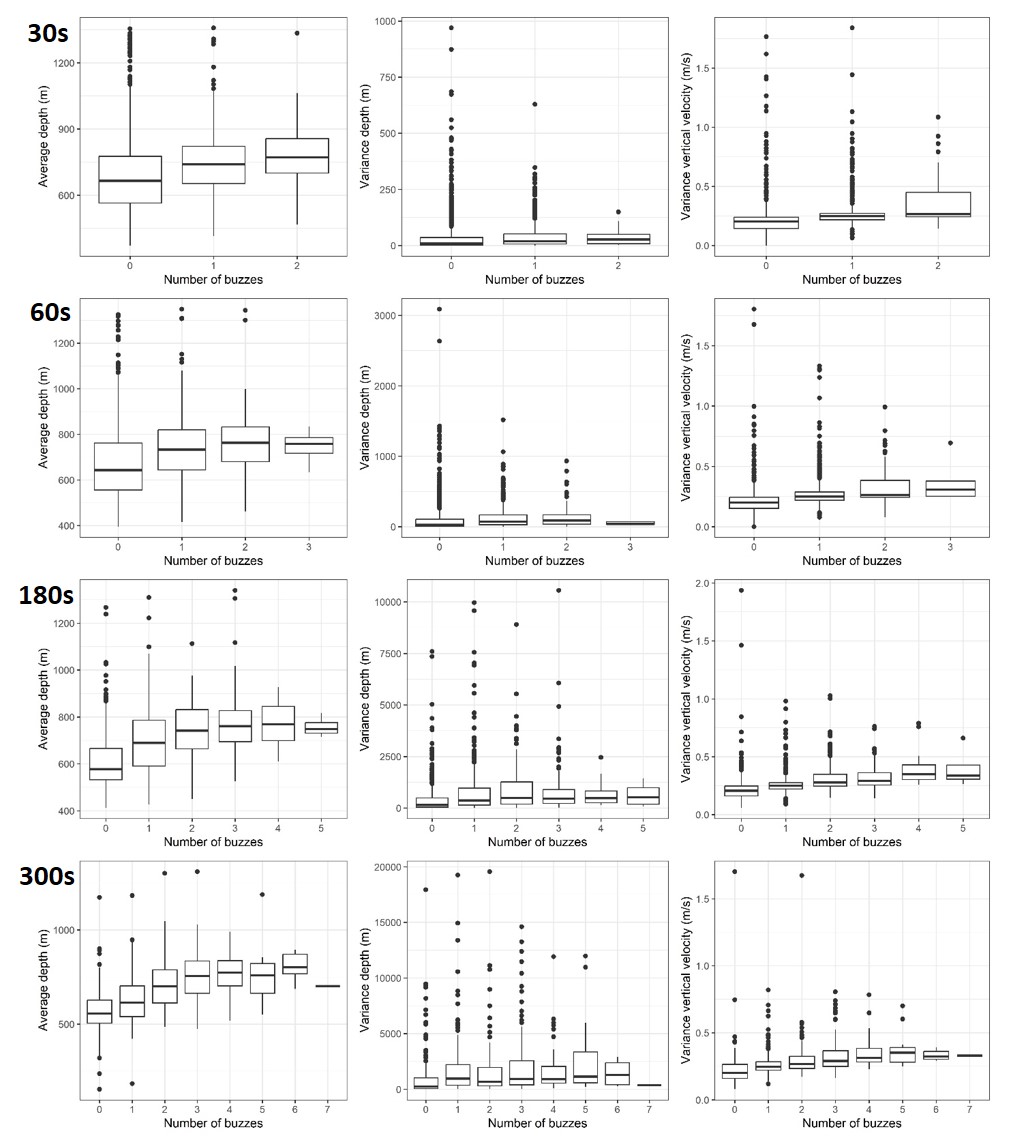
**

**Fig. A2.** Distribution of dive metrics per number of buzzes for each segment duration using only data from the bottom dive phase. The horizontal line represents the median, the box represents the 25th and 75th percentiles, the whiskers represent the extreme values within 1.5 times the length of the box, and dots beyond the end of the whiskers are outlier points..

**
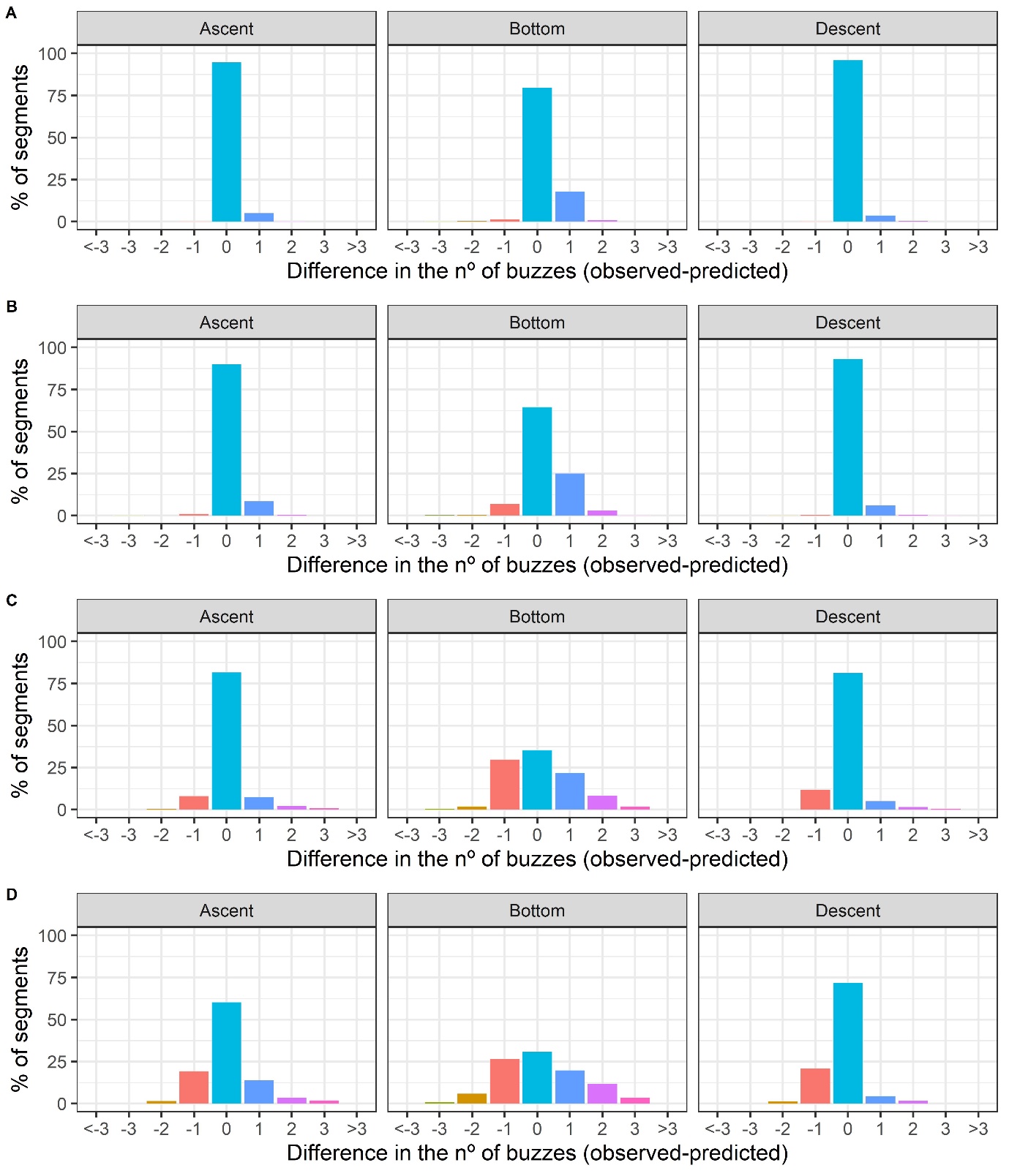
Fig. A3.** Differences between the total number of observed and predicted buzzes per segment at different dive phases for four segment durations: **A)** 30 s, **B)** 60 s, **C)** 180 s and **D)** 300 s, shown as a percentage (%) of the segments. Results obtained from the sensitivity analysis with 100 model runs.


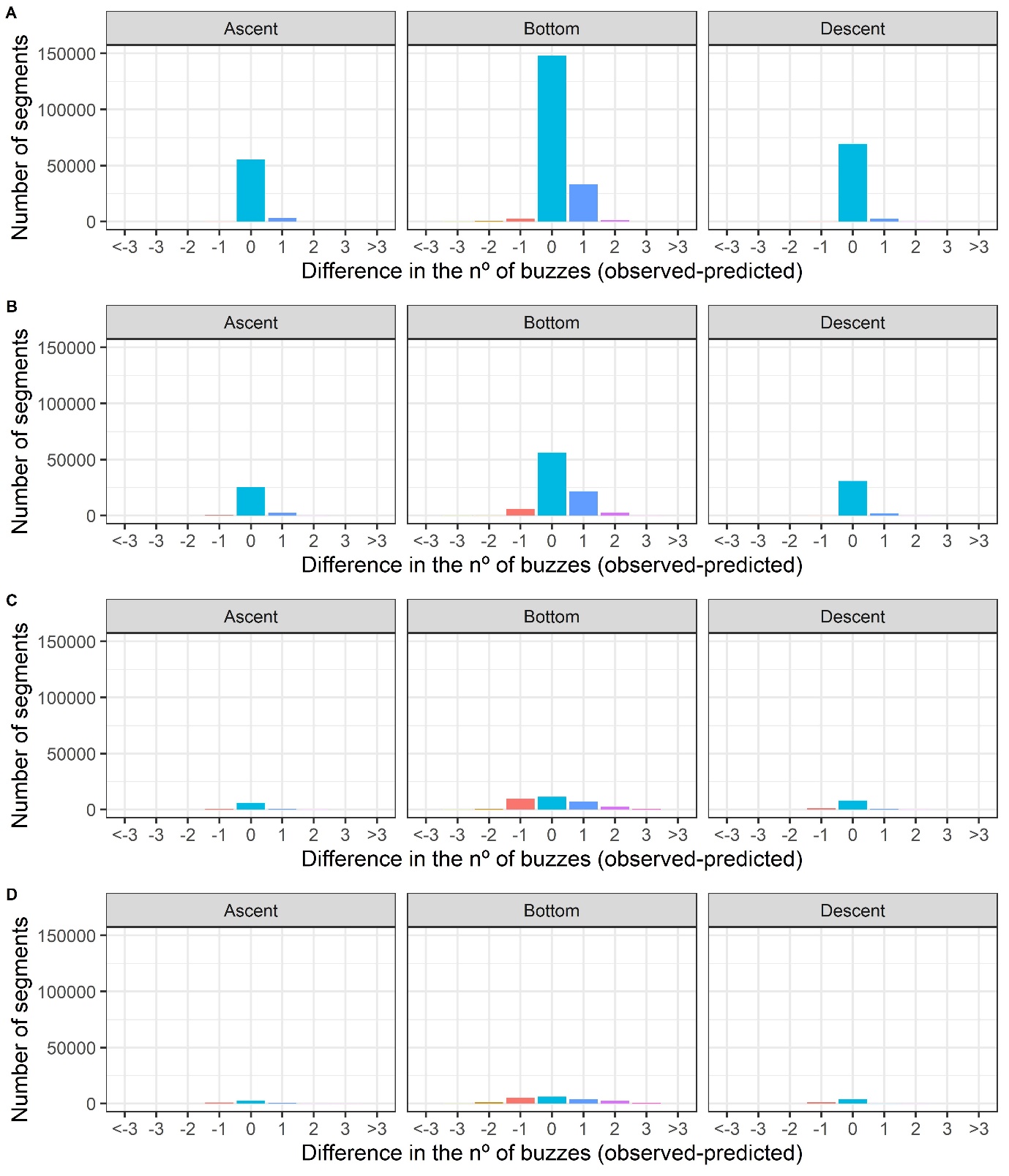


**Fig. A4.**- Differences between the total number of observed and predicted buzzes per segment at different dive phases for four segment durations: **A)** 30 s, **B)** 60 s, **C)** 180 s and **D)** 300 s, shown as the total number of segments. Results obtained from the sensitivity analysis with 100 model runs.


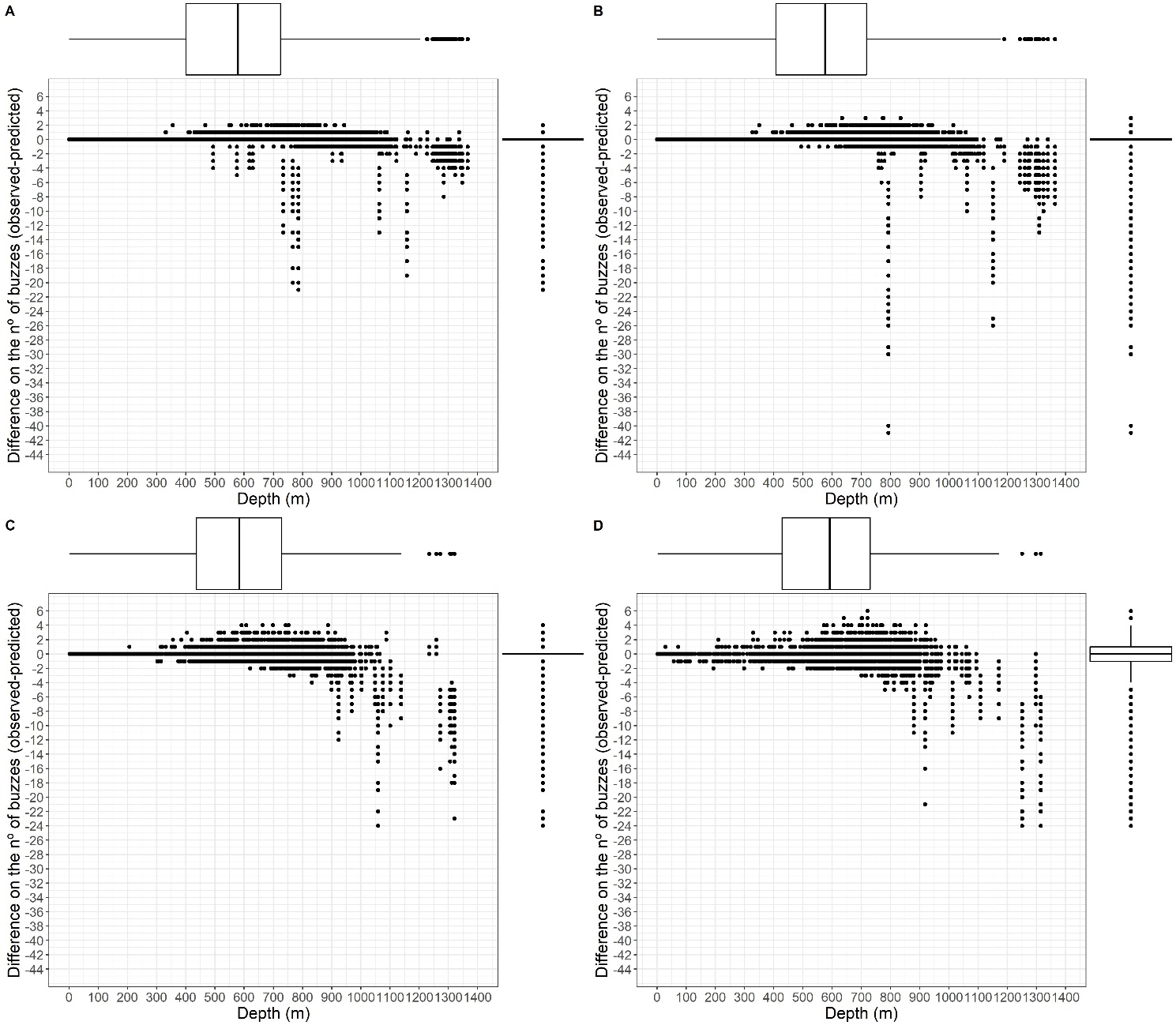


**Fig. A5.** Differences between the total number of observed and predicted buzzes per segment at each depth for four segment durations: **A)** 30 s, **B)** 60 s, **C)** 180 s and **D)** 300 s. Results obtained from the sensitivity analysis with 100 model runs.
